# Impact of migraine on the clinical presentation of insomnia: a population-based study

**DOI:** 10.1186/s10194-018-0916-5

**Published:** 2018-09-14

**Authors:** Jiyoung Kim, Soo-Jin Cho, Won-Joo Kim, Kwang Ik Yang, Chang-Ho Yun, Min Kyung Chu

**Affiliations:** 1 Department of Neurology, BioMedical Research Institute, Pusan National University Hospital, Pusan National University School of Medicine, Busan, South Korea; 2 Department of Neurology, Dongtan Sacred Heart Hospital, Hallym University College of Medicine, Hwaseong, South Korea; 3 Department of Neurology, Gangnam Severance Hospital, Yonsei University College of Medicine, Seoul, South Korea; 4 Sleep Disorders Center, Department of Neurology, Soonchunhyang University College of Medicine, Cheonan Hospital, Cheonan, South Korea; 5 Clinical Neuroscience Center, Department of Neurology, Seoul National University Bundang Hospital, Seongnam, South Korea; 6 Department of Neurology, Severance Hospital, Yonsei University College of Medicine, 50-1 Yonsei-ro, Seodaemoon-gu, Seoul, Republic of Korea

**Keywords:** Clinical presentation, Headache, Insomnia, Insomnia symptom, Migraine

## Abstract

**Background:**

Insomnia and migraine are closely related; insomnia aggravates migraine symptoms. This study was conducted to investigate the impact of migraine on the clinical presentation of insomnia symptoms.

**Methods:**

The data of the Korean Headache-Sleep Study (KHSS) were used in the present study. The KHSS is a nation-wide cross-sectional population-based survey regarding headache and sleep in Korean adults aged 19 to 69 years. If a participant’s Insomnia Severity Index (ISI) score ≥ 10, she/he was classified as having insomnia. The clinical presentation of insomnia symptoms was assessed using total and subcomponent scores of the ISI.

**Results:**

Of 2695 participants, 290 (10.8%) and 143 (5.3%) individuals were assigned as having insomnia and migraine, respectively. The proportions of migraine (12.8% vs. 4.4%, *p* <  0.001) and non-migraine headache (59.0% vs. 39.9%, *p* <  0.001) were higher among individuals with insomnia compared to those without insomnia. Among participants with insomnia, total ISI scores were not significantly different among participants with migraine, non-migraine, and non-headache [median and interquartile range: 13.0 (11.0–17.5) vs. 13.0 (11.0–17.5) vs. 12.0 (11.0–16.0), *p = 0.245*]. ISI scores for noticeability of sleep problems to others were significantly higher among participants with migraine [3.0 (2.0–4.0) vs. 2.0 (2.0–3.0), *p = 0.011*] and non-migraine headache [3.0 (2.0–4.0) vs. 2.0 (2.0–3.0), *p = 0.001*] compared to those without headache history. Other ISI subcomponent scores did not significantly differ between headache status groups.

**Conclusions:**

Participants with insomnia had an increased risk of migraine and non-migraine headache compared to those without insomnia. Among participants with insomnia, overall insomnia severity was not significantly influenced by the headache status.

## Background

Migraine is a common neurological disorder and affects 5–15% of the general population [1]. Owing to its disabling symptoms, migraineurs encounter disability and decreased quality of life [2]. Even in periods without migraine symptoms, migraineurs may have a fear of developing a headache because migraine attacks often cause a failure to perform social obligations at school, the workplace, or at home [3]. Sleep disturbances are common complaints among migraineurs [4–6]. Individuals with migraine or headache with sleep disturbances often encounter more severe symptoms and decrease quality of life [5, 7, 8].

Insomnia is another disorder with high prevalence, affecting 10–30% of the general population [9]. Insomnia is associated with hypertension, coronary heart disease, and diabetes, among others [10]. Furthermore, insomnia in the working age population is one of the factors that cause a decrease in productivity [11]. Therefore, insomnia is an important public health problem like migraine.

Migraine and insomnia exhibit a strong relationship. Cross-sectional studies have persistently demonstrated a significant comorbidity for these two disorders in clinical and population-based studies [12]. Two longitudinal studies using a single dataset show a bidirectional comorbidity of migraine and insomnia. Individuals have an increased risk of developing migraine 11 years after the onset of insomnia and vice versa [13, 14]. The risk for developing insomnia increases in patients with an increased migraine headache frequency, and the risk for developing migraine was positively correlated with severe insomnia. Such a strong bidirectional comorbidity suggests shared pathophysiological mechanisms [15].

According to previous studies, migraineurs have an increased risk of developing insomnia compared to patients suffering from non-migraine headache and healthy subjects. Furthermore, migraineurs with insomnia present with a higher headache frequency and increased headache intensity compared to those without insomnia [8]. Nevertheless, information about the impact of migraine on the prevalence and clinical presentation of insomnia in a population-based sample is currently limited. We hypothesized that migraine affects the prevalence and clinical presentation of insomnia symptoms. The purposes of the present study were to investigate 1) the prevalence of migraine and insomnia, 2) the impact of migraine on the prevalence of insomnia, and 3) the impact of migraine on the clinical presentation of insomnia in a general population-based sample.

## Methods

### Study population and survey process

The data of the Korean Headache-Sleep Study (KHSS) were used in the present study. The KHSS was a nation-wide, cross-sectional survey regarding headache and sleep disorder among Korean adults aged 19 to 69 years. It also included items regarding symptoms of anxiety and depression. The study design, methods, and process were described in detail previously [6]. In brief, the KHSS adopted a two-stage clustered random sampling method for all Korean territories except Jeju-do. This method sampled participants proportionally to the population distribution and socioeconomic status. Trained interviewers conducted the survey by face-to-face interviews using a questionnaire. All trained interviewers were employees of Gallup Korea and had previous experience in social surveys. Data collection of the KHSS was performed from November 2011 to January 2012. The KHSS was approved by the Institutional Review Board and Ethics Committee of Hallym University Sacred Heart Hospital (IRB No. 2011-I077). Written informed consent was obtained from all participants.

### Migraine assessment

Diagnosis of migraine was based on criteria A to D for migraine without aura in the third edition beta version of the International Classification of Headache Disorders (ICHD-3 beta; code 1.1: A, 5 or more attacks in a lifetime; B, attack duration of 4–72 h; C, any 2 of the 4 typical headache characteristics [i.e., unilateral pain, pulsating quality, moderate-to-severe pain intensity, and aggravation by routine physical activity]; and D, attacks associated with at least one of the following: nausea, vomiting, or both photophobia and phonophobia) [16]. We did not distinguish between migraine without aura (code 1.1) and migraine with aura (code 1.2). Therefore, migraine included both migraine with aura and migraine without aura. Our survey method has been reported to have a sensitivity of 75.0% and a specificity of 88.2% [17].

### Non-migraine headache assessment

Participants that experienced more than one minute of headache in the last twelve months but did not satisfy the criteria for migraine diagnosis were classified as a distinct non-migraine headache group.

### Insomnia assessment

Insomnia was evaluated by using the Insomnia Severity Index (ISI), which is a self-report questionnaire with the following seven items: difficulties in sleep onset, difficulties in sleep maintenance, early awakening in the morning, sleep dissatisfaction, interference of sleep problems with daily functioning, noticeability of sleep problems to others, and worries caused by the sleep problems. This index measures the individual’s perceptions of their sleep problem by evaluating the severity of the insomnia problems within the last two weeks. The total ISI score ranges from 0 to 28 [18]. Participants with a total ISI score of 10 or more were classified in our study as suffering from insomnia according to a previous epidemiological study [19]. Additionally, we investigated whether the sleep was usually non-refreshing. We asked participants to choose from the categories none, mild, moderate, severe, and very severe and graded each response as 0, 1, 2, 3, and 4, respectively.

### Statistical analyses

The Kolmogorov-Smirnov test was used to evaluate the normality of the distribution. After normality was confirmed, Student’s t-test or analysis of variance was used to compare continuous variables. If normality was not confirmed, the Mann-Whitney *U* test was used to compare differences between two independent groups. For comparison of ordinal variables among more than three groups, we used Kruskal-Wallis test. To adjust for multiple testing, *p-values* were calculated using the Bonferroni post hoc test. Categorical variables were compared using the chi-square test. The significance level was set at *p* < 0.05 for all analyses. Statistical analyses were performed using the Statistical Package for Social Sciences 22.0 (SPSS 22.0; IBM, Armonk, NY, USA).

## Results

### Survey

**Fig. 1:**
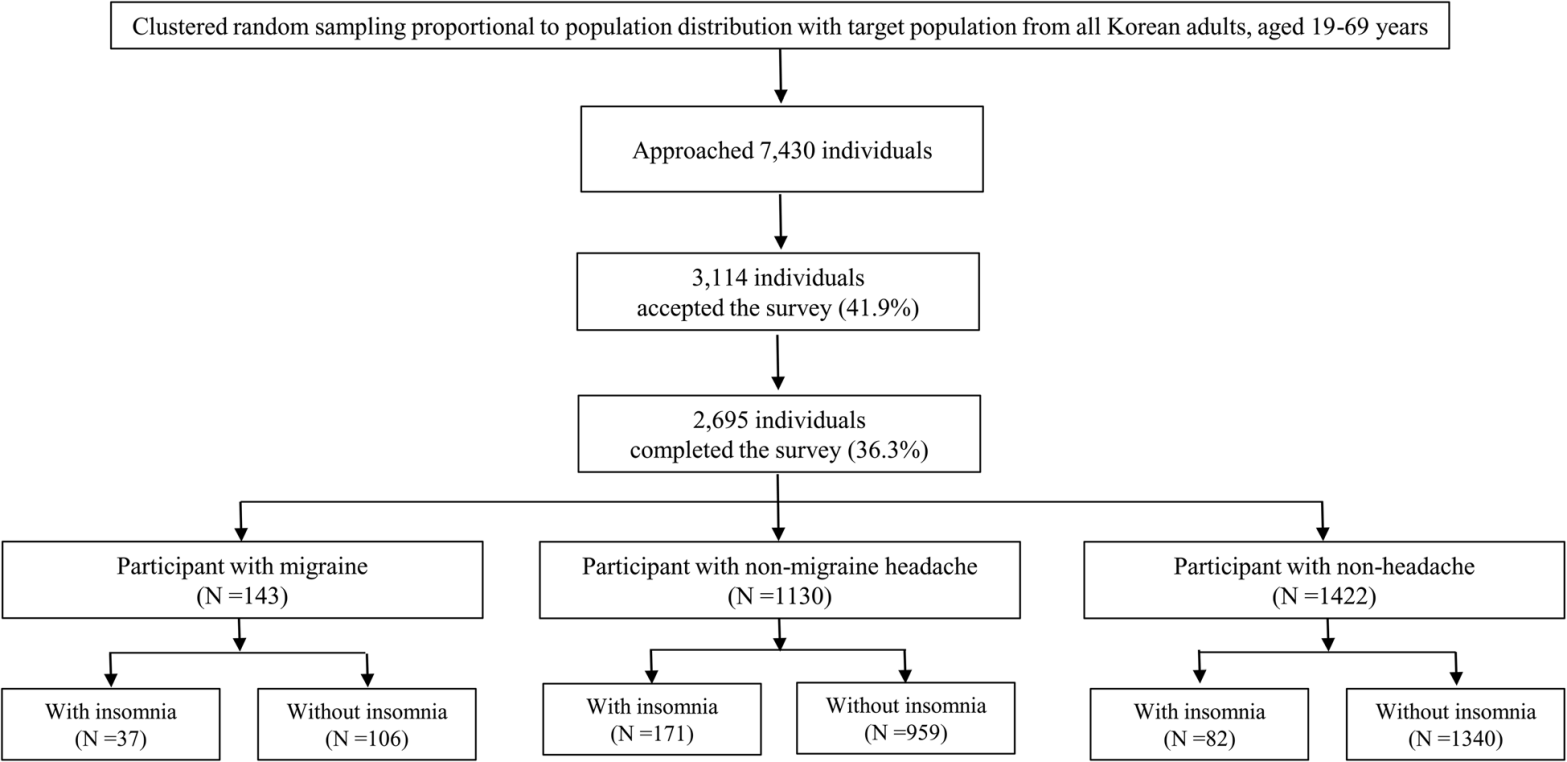
Flowchart depicting the participation of subjects in the KHSS

During the survey, interviewers contacted 7430 individuals, and 3144 permitted to participate. Of those, 449 individuals waived participation, and thus 2695 participants completed the whole survey (cooperation rate of 36.3%; Fig. 1). The distributions of age, sex, size of the residential area, and education level of KHSS participants was not significantly different from those of the Korean general population (Table 1).

**Table 1:** Sociodemographic characteristics of survey participants, the total Korean population, and cases identified as having migraine, non-migraine headache, and insomnia

	Survey participants N (%)	Total population N (%)	*P*	Migraine N, % (95% CI)	Non-migraine headache N, % (95% CI)	Insomnia N, % (95% CI)
Gender						
Men	1345 (49.3)	17,584,365 (50.6)	*0.854* ^*a*^	36, 2.7 (1.8–3.5)	471, 35.0 (32.5–37.6)	117, 8.7 (7.2–10.2)
Women	1350 (50.7)	17,198,350 (49.4)		107, 7.9 (6.5–9.4)	659, 48.8 (46.2–51.5)	173, 12.8 (11.0–14.6)
Age						
19–29	542 (20.5)	7,717,947 (22.2)	*0.917* ^*a*^	25, 4.5 (2.7–6.2)	231, 42.6 (38.4–46.8)	59, 10.9 (8.3–13.5)
30–39	604 (21.9)	8,349,487 (24.0)		42, 7.0 (4.9–9.1)	269, 44.5 (40.6–48.5)	53, 8.8 (6.5–11.0)
40–49	611 (23.1)	8,613,110 (24.8)		39, 6.5 (4.5–8.4)	277, 45.3 (41.4–49.3)	66, 10.8 (8.3–13.3)
50–59	529 (18.9)	6,167,505 (17.7)		22, 4.1 (2.4–5.9)	204, 38.6 (34.4–42.7)	63, 11.9 (9.1–14.7)
60–69	409 (15.6)	3,934,666 (11.3)		15, 3.9 (2.0–5.7)	149, 36.4 (31.7–41.1)	49, 12.0 (8.8–15.1)
Size of residential area						
Large city	1248 (46.3)	16,776,771 (48.2)	*0.921* ^*a*^	76, 6.1 (4.8–7.5)	525, 42.1 (39.3–44.8)	136, 10.9 (9.2–12.6)
Medium-to-small city	1186 (44.0)	15,164,345 (43.6)		48, 4.0 (2.9–5.2)	488, 41.1 (38.3–44.0)	125, 10.5 (8.8–12.3)
Rural area	261 (9.7)	2,841,599 (8.2)		19, 7.4 (4.2–10.6)	117, 44.8 (38.8–50.9)	29, 11.1 (7.3–14.9)
Education level						
Middle school or less	393 (14.9)	6,608,716 (19.0)	*0.752* ^*a*^	22, 5.5 (4.2–7.7)	156, 42.0 (37.1–46.9)	62, 15.8 (12.2–19.4)
High school	1208 (44.5)	15,234,829 (43.8)		60, 5.0 (3.8–6.3)	502, 41.6 (38.8–44.4)	116, 9.6 (7.9–11.3)
College or more	1068 (39.6)	12,939,170 (37.2)		60, 5.6 (4.3–7.0)	457, 42.8 (40.0–45.8)	109, 10.2 (8.4–12.0)
Not responded	26 (1.0)			1, 3.8 (0.0–11.8)	6, 23.1 (5.7–40.4)	3, 11.5 (0.0–24.7)
Total	2695 (100.0)	34,782,715 (100.0)		143, 5.3 (4.5–6.2)	1130, 41.9 (40.0–43.8)	290, 10.8 (9.6–11.9)

^*a*^Comparison of sex, age group, size of residential area, and educational level distributions between the sample in the present study and the total population of Korea

*N,* number; *CI,* confidence interval

### Prevalence of insomnia and migraine

Of the 2695 participants, 290 (10.8%) participants reported an ISI score ≥ 10 and were classified as having insomnia. The ISI score of all participants was [2.0 (1.0–5.0), median and interquartile range]. Of the 1273 (47.2%) participants, who reported that they experienced at least one attack of headache during the last year, 143 (5.3%) participants were classified as having migraine. Therefore, 1130 (41.9%) were classified as having non-migraine headache (Table 1).

### Prevalence of migraine and non-migraine headache according to the presence of insomnia

**Fig. 2:**
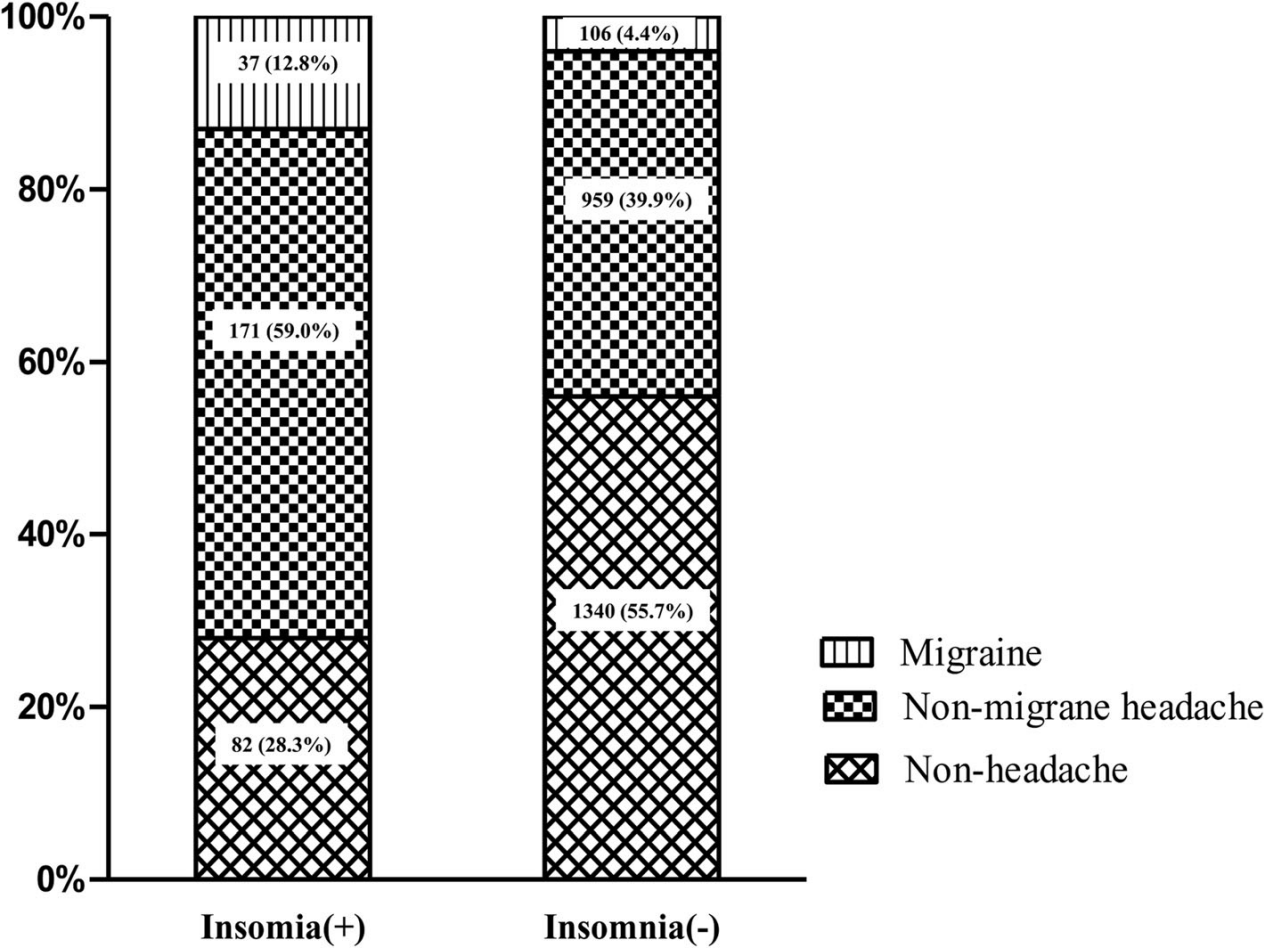
Comparison of headache type according to the presence of insomnia

Among the 290 participants with insomnia, 37 (12.8%), 171 (59.0%), and 82 (28.3%) participants were classified as having migraine, non-migraine headache, and non-headache, respectively. The prevalence of migraine (12.8% vs. 4.4%, *p* < 0.001) and non-migraine headache (59.0% vs. 39.9%, *p* < 0.001) was significantly higher among participants with insomnia compared to that in participants without insomnia (Fig. 2).

### Total and subcomponent ISI scores among participants with insomnia according to the headache status

In the 290 participants with insomnia, the total ISI score was not significantly different among migraine, non-migraine headache, and non-headache groups. A further analysis of the seven ISI subcomponent scores revealed that only the categories interference with daily functioning and noticeability of sleep problems to others exhibited significantly different scores among the three headache groups. Furthermore, our additional parameter non-refreshing sleep showed significant score differences among these groups. Post hoc analyses revealed that the scores for noticeability of sleep problems to others, and non-refreshing sleep were significantly higher in participants of the non-migraine headache group than those in the non-headache group and score for noticeability of sleep problems to others was significantly higher in participants of the migraine group than those in the non-headache group. However, subcomponent for interference with daily functioning did not show significance in post hoc analysis (Table 2).

**Table 2:** Total ISI and its subcomponent scores among participants with insomnia in relation to the headache status

	Migraine (*N* = 37)	Non-migraine headache (*N* = 171)	Non-headache (*N* = 82)	*P*
Total ISI score	13.0 (11.0–17.5)	13.0 (11.0–17.0)	12.0 (11.0–16.0)	*0.245*
Falling asleep	2.0 (1.0–3.0)	2.0 (1.0–3.0)	2.0 (1.0–3.0)	*0.796*
Staying asleep	2.0 (1.0–3.0)	2.0 (1.0–3.0)	2.0 (1.0–3.0)	*0.671*
Early awakening	2.0 (1.0–3.0)	2.0 (1.0–3.0)	2.0 (1.0–3.0)	*0.303*
Satisfaction	4.0 (3.5–4.0)	4.0 (3.0–5.0)	4.0 (3.0–4.0)	*0.245*
Interference	3.0 (3.0–4.0)	3.0 (2.0–4.0)	3.0 (2.0–3.0)	*0.032*
Noticeability	3.0 (2.0–4.0)^a^	3.0 (2.0–4.0)^a^	2.0 (2.0–3.0)	*0.002*
Worry	3.0 (2.0–4.0)	3.0 (2.0–4.0)	3.0 (2.0–3.0)	*0.364*
Non-refreshing sleep^b^	2.0 (2.0–3.0)	3.0 (2.0–4.0)^a^	2.0 (1.0–3.0)	*0.001*

Variables are presented as median (interquartile range)

Kurskal-Wallis test was used to compare among three groups

^a^Significantly higher in the post hoc analysis compared to the non-headache group

^b^Non-refreshing sleep score is not included in the total ISI score

### Prevalence of insomnia according to the headache status

**Fig. 3:**
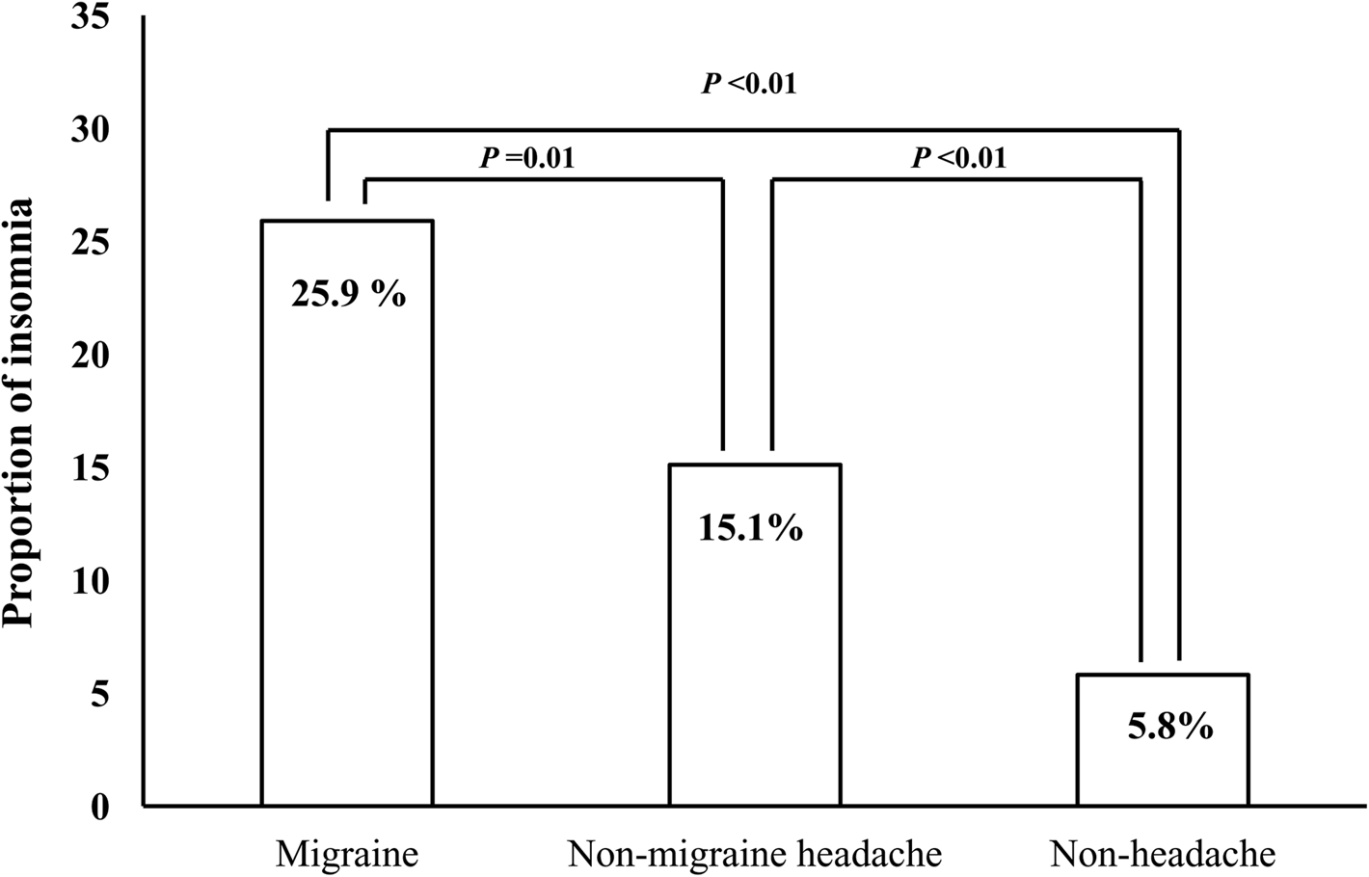
Comparison of the prevalence of insomnia according to headache type

Insomnia prevalence among participants with migraine, non-migraine headache, and non-headache was 25.9%, 15.1%, and 5.8%, respectively (Fig. 3). The prevalence of insomnia among migraineurs was significantly higher compared to participants with non-migraine headache (*p* = 0.001) and non-headache (*p* < 0.001).

### Headache frequency, headache intensity, and impact of headache according to the presence of insomnia among participants with migraine and non-migraine headache

The Visual Analogue Scale (VAS) score for headache intensity (median [interquartile range], 7.0 [5.0–9.0] vs. 6.0 [5.0–7.0]; *p* = 0.003) and the Headache Impact Test-6 (HIT-6; 60.0 ± 9.5 vs. 52.3 ± 8.4; *p* < 0.001) score were significantly higher in migraineurs with insomnia compared to those in migraineurs without insomnia. Headache frequency per month was discernibly different according to the presence of insomnia without reaching statistical significance (5.7 ± 8.2 vs. 3.2 ± 5.4, *p* = 0.093). Among participants with non-migraine headache, headache frequency per month, VAS score for headache intensity, and HIT-6 score were significantly higher when insomnia was present (Table 3).

**Table 3:** Headache frequency, VAS score of headache intensity, and impact of headache in relation to the presence of insomnia among participants with migraine and non-migraine headache

	Migraine with insomnia (*N* = 37)	Migraine without insomnia (*N* = 106)	*P*	Non-migraine headache with insomnia (*N* = 171)	Non-migraine headache without insomnia (*N* = 959)	*P*
Headache frequency	5.7 ± 8.2	3.2 ± 5.4	*0.093*	3.7 ± 6.8	2.1 ± 4.9	*0.004*
VAS score^a^	7.0 (5.0–9.0)	6.0 (5.0–7.0)	*0.003*	5.0 (4.0–7.0)	5.0 (3.0–6.0)	*< 0.001*
HIT-6 score	60.0 ± 9.5	52.3 ± 8.4	*< 0.001*	50.1 ± 8.6	44.4 ± 6.7	*< 0.001*

*VAS* Visual Analogue Scale, *HIT-6* Headache Impact Test-6

^a^Variable is analyzed by Mann–Whitney U test and shown as a median (interquartile range)

## Discussion

The key findings of the present study were as follows: 1) The prevalence of migraine and non-migraine headache was significantly higher among participants with insomnia compared to those without insomnia; 2) Among participants with insomnia, the total ISI score was not significantly different among migraine, non-migraine headache, and non-headache groups and 3) Among participants with migraine, the prevalence of insomnia was higher than participants with non-migraine headache and non-headache. Migraine symptoms exacerbated with the presence of insomnia.

There is limited information available about the impact of insomnia on the clinical presentation of headache. It has been reported that in individuals with headache or migraine, those with insomnia present an increased symptom severity compared to those without [13]. Nevertheless, information regarding the impact of headache or migraine on the clinical presentation of insomnia is currently scarce. Our study is the first report in a population-based setting that individuals with insomnia have an increased risk of suffering from migraine and non-migraine headache. Furthermore, the insomnia severity as reflected by the total ISI score did not differ among headache status groups in individuals with insomnia (Table 2). These findings suggest that the headache status does not influence the overall severity of insomnia among affected individuals. In contrast, insomnia has a significant impact on the clinical presentation of migraine. Migraineurs with insomnia experienced a higher headache intensity and impact of headache (HIT-6 score) compared to migraineurs without insomnia (Table 3).

What could be the underlying mechanism for the difference between the impact of migraine on insomnia severity and the impact of insomnia on migraine severity? It is possible that their distinct anatomy and pathophysiology contribute to the contrast findings. Hypothalamus has been understood to play key regulatory roles both for migraine and sleep controls. The supraoptic nucleus in anterior region plays a key role in the regulation of sleep and arousal. Pain perception was regulated by arcuate nucleus in tuberal region [20]. Although supraoptic nucleus and arcuate nucleus are located nearby in hypothalamus, they have distinctive anatomical locations. A recent functional magnetic resonance imaging study showed that posterior hypothalamic activation was noted during the acute migraine stage [21]. Orexinergic system acts a regulatory role both in sleep and pain. Orexin-A and orexin-B were synthesized in hypothalamus and promote arousal. Nevertheless, they do different roles in pain modulation. Orexin-A is able to inhibit neurogenic dural vasodilation via activation of the orexin receptor type 1, resulting in inhibition of prejunctional release of calcitonin-gene related peptide from trigeminal neurons [22]. In contrast, orexin-B increases the A and C-fibre responses to dural electrical stimulation as well as spontaneous activity [23].

Among the ISI subcomponents, the score for noticeability of sleep problems to others was significantly higher among participants with migraine and non-migraine headache compared to participants without headache. Furthermore, the score for non-refreshing sleep were significantly higher among participants with non-migraine headache compared to participants without headache (Table 2). In contrast, other subcomponent scores including difficulties in sleep onset, difficulties in sleep maintenance, early awakening, sleep dissatisfaction, and worries caused by the current sleep problems were not significantly different. The distinct association of the headache status with only certain subcomponents of insomnia suggests that certain subcomponents are distinctive from other subcomponents. Further, our findings are in agreement with the previous findings that nonrestorative sleep (NRS) is a distinctive subtype of insomnia from other subtypes of insomnia. These studies showed that individuals with NRS was more frequently associated with daytime impairment than individuals with difficulty initiation of sleep (DIS), difficulty maintaining sleep (DMS) and early morning awakening (EMA) [24–27]. While NRS is reported to be associated with longer sleep latency, shorter sleep duration, increased alpha activity during non-rapid eye movement sleep, longer duration of cyclic alternating patterns and chronic pain condition, information on the differences in pathophysiology of NRS from other subtypes of insomnia is currently sparse [25, 28–30]. Therefore, more research on pathophysiology of NRS is needed for better management of NRS.

In the present study, we used the ISI to assess insomnia. This psychometric scale includes items for difficulty in falling asleep, difficulty in staying asleep, and early awakening which enables the classification of insomnia subtypes [18]. Although we did not identify insomnia cases by interviews based on International Classification of Sleep Disorders, 3rd edition or the Diagnostic and Statistical Manual of Mental Disorders, 5th edition criteria which are the ‘gold standard’ for the evaluation of insomnia, we are convinced that we successfully evaluated insomnia because the insomnia prevalence in the present study is in a similar range found in Asian countries by previous studies [29, 31, 32].

The present study has some limitations. First, we could not evaluate whether participants of this study underwent pharmacological or non-pharmacological treatment for insomnia. Second, as this is a cross-sectional study, we could not investigate changes in insomnia symptoms before and after migraine treatment. Third, the overall response rate was not high in our study. However, we used a two-stage clustered random sampling, proportional to the population distribution of Korea. Therefore, the distribution of age, sex, size of residential area, and educational level of our participants was similar to those of the Korean general population. In addition, the prevalence of migraine, non-migraine and insomnia in the KHSS were similar to that of previous studies [17, 33]. Despite these limitations, there are several strengths in the present study. First, the impact of migraine on the severity and clinical presentation of insomnia symptoms as well as on the prevalence of insomnia was evaluated. Second, the present study also evaluated the impact of insomnia on the clinical presentation migraine. Third, the distributions of age, sex, size of the residential area, and education level in this study’s participants were not significantly different from the general population in Korea. The results of this study seem to appropriately reflect the characteristics of the general Korean population.

## Conclusions

Subjects with insomnia have an increased prevalence of migraine and vice versa in a population-based setting. Although insomnia is associated with increased headache frequency and severity among migraineurs, insomnia severity is, apart from some subcomponents, not significantly influenced by the presence of migraine. Our findings indicate that migraine and insomnia affect each other but their asymmetric causal relationship needs further investigation in future studies.

## Abbreviations

CI: Confidence Interval
DIS: Difficulty Initiating Sleep
DMS: Difficulty Maintaining Sleep
DSM: Diagnostic and Statistical Manual of Mental Disorders
EMA: Early Morning Awakening
HIT-6: Headache Impact Test-6
ICHD: International Classification of Headache Disorders
ICSD: International Classification of Sleep Disorders
ISI: Insomnia Severity Index
KHSS: Korean Headache-Sleep Study
NRS: Non-Restorative Sleep
VAS: Visual Analogue Scale
